# Study of Graphene Oxide and Silver Nanowires Interactions and Its Association with Electromagnetic Shielding Effectiveness

**DOI:** 10.3390/ijms252413401

**Published:** 2024-12-13

**Authors:** Mila Milenković, Warda Saeed, Muhammad Yasir, Dusan Sredojević, Milica Budimir, Andjela Stefanović, Danica Bajuk-Bogdanović, Svetlana Jovanović

**Affiliations:** 1 Vinča Institute of Nuclear Sciences-National Institute of the Republic of Serbia, University of Belgrade, Mike Petrovića Alasa 12-14, Vinča, 11351 Belgrade, Serbia; mila.milenkovic@vin.bg.ac.rs (M.M.); dusredo@vin.bg.ac.rs (D.S.); budimir@vin.bg.ac.rs (M.B.);; 2 Division of Microrobotics and Control Engineering, Department of Computing Science, Carl von Ossietzky Universität Oldenburg, 26129 Oldenburg, Germany; warda.saeed@uni-oldenburg.de; 3 Faculty of Physical Chemistry, University of Belgrade, Studentski trg 12-16, 11158 Belgrade, Serbia; danabb@ffh.bg.ac.rs

**Keywords:** graphene, silver nanowires, density functional theory, electromagnetic shielding

## Abstract

Technological development has led to the need for materials able to block electromagnetic waves (EMWs) emitted from various devices. EMWs could negatively affect the working performance and lifetime of multiple instruments and measuring devices. New EMW shielding materials are being developed, while among nanomaterials, graphene-based composites have shown promising features. Herein, we have produced graphene oxide (GO), silver nanowires (AgNWs) composites, by varying the mass ratios of each component. UV-Vis, infrared, Raman spectroscopies, and thermogravimetric analysis proved the establishment of the interactions between them. For the first time, the strength and the nature of the interaction between GO sheets with various levels of oxidation and AgNWs were investigated using density function theory (DFT). The interaction energy between ideal graphene and AgNWs was calculated to be −48.9 kcal/mol, while for AgNWs and GO, this energy is almost doubled at −81.9 kcal/mol. The DFT results confirmed the interfacial polarization at the heterointerface via charge transfer and accumulation at the interface, improving the efficacy of EMW shielding. Our results indicated that AgNWs create a compact complex with GO due to charge transfer between them. Charge redistributions in GO-AgNWs composites resulted in an improved ability of the composite to block EMWs compared to GO alone.

## 1. Introduction

Electronic devices emit electromagnetic waves (EMWs), which interfere with electronics due to the interactions of electrons in metal conductors with the electric field of radiation. Electromagnetic interferences (EMIs) can cause electronic devices to malfunction and leak information [1]. Thus, shielding materials are required for both electronics and radiation sources.

The shielding effectiveness (SE) of material is expressed as the loss of power due to the interaction of the incident wave with the material. Power loss is measured in decibels (dB) and is referred to as total shielding effectiveness. The loss can be due to the absorption and is referred to as a dissipation loss (SE_A_) or due to the reflection, known as a reflection loss (SE_R_) [1]. A total shielding effectiveness of 20 dB is equivalent to blocking 99% of incident EMWs, and it is the minimum needed for commercial applications [2].

Nowadays, the most commonly used materials for EMI shielding can be divided into three groups: metals [3], polymers [4], and inorganic non-metallic materials [5]. Metals are conductive but have a heavy weight, low flexibility, high cost, and are corrosive. A possible solution is making metal in the form of films, or metallic wires [6,7], or applying them as a conductive filler in composites. The advantages of polymers are their non-corrosive nature and adjustable density. Both conductive polymers and non-conductive polymers with conductive fillers are being developed. As a filer, conductive polymers, metal nanoparticles, carbon nanostructures [5,8,9], carbon aerogel [10,11], and carbon fiber [12] are studied. However, polymers face several difficulties, including precise control over the shape and characteristics, good filler dispersion, and polymer–polymer interaction. Therefore, inorganic non-metallic materials with the aforementioned benefits and no clear drawbacks have been extensively researched. Carbon-based and ceramic materials make up the majority of it. Carbon-based materials include carbon fiber [13], carbon nanotubes [13,14], graphene [2,15,16], MXene [17], and others.

Demand for thinner, lighter, and more flexible EMI shielding materials is increasing, favoring carbon nanomaterials [18]. Graphene possesses remarkable properties such as high electrical conductivity (10^4^–10^5^ S m^−1^), good flexibility, chemical inertness, mechanical strength, excellent electron mobility (~15,000 cm^2^ V^−1^ s^−1^), tunable electrical property [19,20,21], and great heat conductivity (~5000 Wm^−1^ K^−1^) [22,23,24]. These extraordinary properties make graphene a good candidate for EMI shielding in various electronics [5,25,26,27].

Single-layer graphene produced using chemical vapor deposition showed an EMI SE_T_ of 2.27 dB, where the main shielding mechanism was absorption [4]. The preparation of graphene oxide (GO), using a modified Hummer’s method followed by the reduction (rGO), is one of the most common preparation methods of graphene. Wen et al. reported the shielding effectiveness of graphene/paraffin wax composites to be higher than 20 dB, with 20 wt.% of graphene [28]. In another study, Chen et al. prepared graphene/epoxy composites and obtained a shielding effectiveness of 21 dB for the 15 wt.% loading of graphene [29]. Combining graphene with metal nanostructures, such as silver nanowires (AgNWs), is one approach to increase electrical conductivity and EMI SE. AgNWs create highly conducting composites, such as thin films, sandwich structures, foams, and fibers [30,31,32,33]. However, with their high reactivity and surface area, they oxidize and react with atmospheric S oxides, making them unstable in the air. AgNWs must therefore be isolated from contact with air or water [34].

In this study, we prepared GO and AgNWs composites by changing the mass ratios between the two nanomaterials. We investigated the interaction between these nanomaterials using UV-Vis, Raman, and infrared spectroscopic techniques. The thermal stability of composites was studied as well. Considering that the GO and rGO are non-uniformly coated with hydroxyl and epoxy groups at the surface, including the regions with no basal groups (ideal-like graphene), we constructed several GO clusters to model the interactions with AgNWs. For the first time, theoretical modeling of interactions between the surfaces of AgNWs and GO at different levels of oxidation was employed and connected to the ability of composites to block the propagation of electromagnetic waves at frequencies in the range of 8–12 GHz. Namely, the effects of the content of GO and AgNWs in composite on the shielding effectiveness and mechanism were investigated using a vector network analyzer. We observed that the increased mass content of AgNWs improves EMI SE. Previous studies were focused on AgNW’s improvement of electrical conductivity of GO-AgNWs composites, which consequently amplifies the shielding efficiency of composites [35,36,37]. Therefore, we have examined and observed two different nanomaterials, their interactions, and the effects of these interactions on incident EMWs. Considering the results of experimental and theoretical studies, we observed the charge transfer from the graphene core to the AgNWs, leading to the enhancement of EMI SE at heterointerfaces.

## 2. Results and Discussion

### 2.1. Investigation of GO-AgNWs Interactions

**Figure 1:**
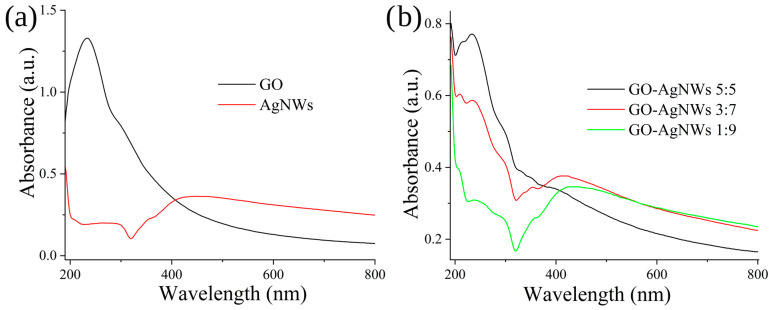
UV-Vis spectra of GO and AgNWs (**a**), GO-AgNWs 5:5, GO-AgNWs 3:7, and GO-AgNWs 1:9 (**b**).

UV-Vis spectra of GO, AgNWs, and composites are shown in Figure 1a,b, and in Figure S1 (Supporting Information). The main absorption band in the GO spectrum (Figure 1a) is centered at 234 nm while the shoulder band with a lower intensity is around 310 nm. The first one is associated with the π→π* electronic transition of aromatic Csp^2^-Csp^2^ while the shoulder band is a result of electronic transition n→π transitions of C=O bonds. In the UV-Vis spectrum of AgNWs (Figure 1a), bands at 355 nm and 420 nm are observed. The peak at 355 nm stems from longitudinal plasmon resonance absorption while the second one stems from transversal plasmon resonance absorption [38,39]. In the case of GO–AgNWs 5:5 (Figure 1b), bands at 213, 234, 355, and 410 nm are observed. While the main band assigned to sp^2^ domains in the graphene structure is not shifted, the shoulder band is shifted to 300 nm. In the same spectrum, the band assigned to transversal plasmon resonance is also shifted to 430 nm. Furthermore, a new band at 213 nm is detected. The same band is even more pronounced in the UV-Vis spectrum of GO-AgNWs 4:6 composite (Figure S1, Supporting Information), while the largest changes in spectra of GO-AgNWs 3:7 (Figure 1b) and GO-AgNWs 2:8 (Figure S1, Supporting Information) composites showing shift to 415 and 420 nm, respectively, or 430 nm in the case of GO-AgNWs 1:9 (Figure 1b). The appearance of a new band and the shifts in the existing ones indicate the establishment of interactions between the sp^2^ region and functional groups of GO with the AgNW surfaces.

**Figure 2:**
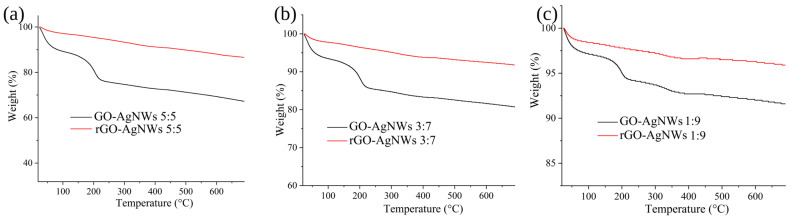
Thermograms of GO-AgNWs 5:5 and rGO-AgNWs 5:5 (**a**), GO-AgNWs 3:7 and rGO-AgNWs 3:7 (**b**), and GO-AgNWs 1:9 and rGO-AgNWs 1:9 (**c**).

Thermal stability and the efficiency of the reduction reaction are investigated using TGA. These results are presented in Figure 2 for GO-AgNWs 5:5, GO-AgNWs 3:7, and GO-AgNWs 1:9 composites, as well as for reduced forms. In Figure S2 (Supporting Information), thermograms for GO-AgNWs 4:6 and GO-AgNWs 2:8 are displayed. All oxidized forms of GO-AgNWs show similar trends, with two degradation steps, first in the range up to 130 °C and second between 130 °C and 250 °C (black curves in Figure 2 and Figure S2, Supporting Information). At temperatures above 250 °C, the weight loss is gradual. The first step is assigned to the evaporation of physically adsorbed water [40], while the second weight loss is attributed to the pyrolysis of oxygen-containing functional groups and the formation of CO_2_ and H_2_O as main decomposition products [41].

Reduced composites (rGO-AgNWs) show improved thermal stability with the reduced total weight loss from 4.37 wt% as measured for GO-AgNWs 1:9 and rGO-AgNWs 1:9 up to 18.64 wt% which is calculated for GO-AgNWs 5:5 and rGO-AgNWs 5:5.

Presented TGA results indicate that the selected reduction procedure removes partially oxygen-containing functional groups from GO in composites and improves the thermal stability of materials.

**Figure 3:**
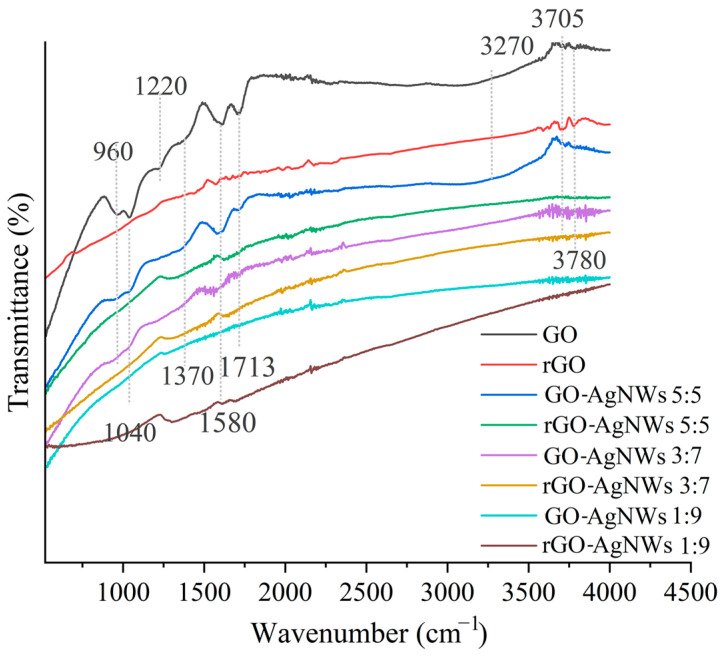
FTIR spectra of GO, rGO, GO-AgNWs 5:5, rGO-AgNWs 5:5, GO-AgNWs 3:7, rGO-AgNWs 3:7, GO-AgNWs 1:9, and rGO-AgNWs 1:9.

FTIR spectroscopy was used to identify the functional groups of GO, rGO, and the GO-AgNWs composites. The results are presented in Figure 3 and Figure S3, Supporting Information.

All non-reduced samples exhibit peaks around 3270 cm^−1^ and 3705 cm^−1^, corresponding to the O-H stretching vibration [42,43]. These peaks are most prominent and clearly defined in the GO sample. The peak at 1713 cm^−1^ is attributed to C=O stretching vibrations, while the peaks at 1580 cm^−1^, 1220 cm^−1^, and 1040 cm^−1^ correspond to C=C and C-O-C bonds, respectively [44,45]. After the reduction in GO to rGO, the O-H and C=O peaks become almost unnoticeable, indicating the removal of oxygen-containing functional groups. In addition, all corresponding peaks in rGO exhibit significantly lower intensities than GO, confirming successful reduction. For GO-AgNWs composites (Figure 3), slight peak shifts and reduced intensities are noticed, suggesting the establishment of the interactions between GO and AgNWs. In the composites with higher AgNW content (Figure 3, GO-AgNWs 3:7, rGO-AgNWs 3:7, GO-AgNWs 1:9, and rGO-AgNWs 1:9), the band assigned to O-H (around 3705 cm^−1^) is still visible but with lowered intensity. In contrast, the bands assigned to C=O and C-O bonds are diminished, suggesting both the reduction in the sample and the successful establishment of interactions between GO and AgNW.

**Figure 4:**
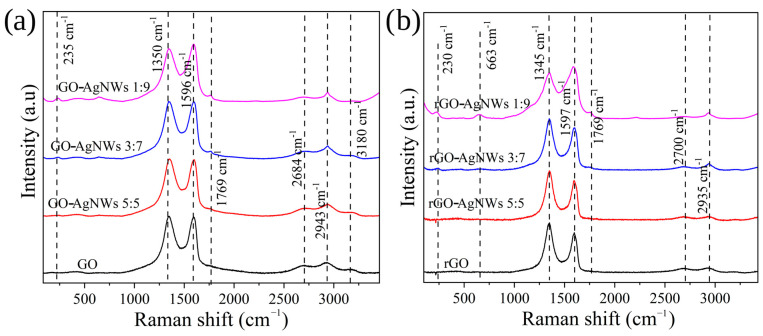
Raman spectra of GO, GO-AgNWs 5:5, GO-AgNWs 3:7, GO-AgNWs 1:9, (**a**) rGO, rGO-AgNWs 5:5, rGO-AgNWs 3:7, and rGO-AgNWs 1:9 (**b**).

Raman spectra of all composites are presented in Figure 4 and Figure S4, Supporting Information. All spectra show bands around 1350 cm^−1^ which is assigned to inherent defect or disorder in sp^2^ domains of graphene sheets and at 1596 cm^−1^ which is a so-called G or graphitic band associated with the phonon vibration of sp^2^ region with E_2g_ symmetry [46,47]. Both D and G bands of composites are redshifted compared to GO and rGO (Table S1, Supporting Information), which was previously assigned to AgNWs adhesion to graphene flakes [48]. The lowering I_D_/I_G_ ratio is proportional to AgNWs content, and these results could be explained as the correction or “healing” of inherent defects [48,49].

Bands between 2700 and 3200 cm^−1^ are also related to graphene-like structures (2D and D + G bands). Additional bands were observed at 235, 663, and 1769 cm^−1^ in spectra of GO-AgNWs 3:7, GO-AgNWs 2:8, and GO-AgNWs 1:9 composites (Figure 4a and Figure S4, Supporting Information), as well as in the Raman spectra of the same composite after reduction (Figure 4b). The band at 235 cm^−1^ stems from Ag–O stretching vibration [50]. It indicates that the PVP molecule is coordinately bonded to the atom of Ag at the nanowire’s surface, and the nonbonding electrons of the O atom in carbonyl functional groups are donating the electron pair [51]. In the spectra of composites GO-AgNWs 5:5, GO-AgNWs 4:6, and GO-AgNWs 3:7, bands characteristic for AgNWs are not observed; it can be concluded that their surface is tightly covered with GO sheets.

Experimental studies (UV-Vis, FTIR, and Raman spectra, Figure 1, Figure 3 and Figure 4) proved that GO and AgNWs create surface interactions. We used DFT to closely understand the nature of the interaction between GO sheets, both sp^2^ region and functional groups with AgNWs, and areas of GO surface that are engaged in the interaction with edges and tips of AgNWs.

### 2.2. Theoretical Investigation of GO-AgNWs Interactions

**Figure 5:**
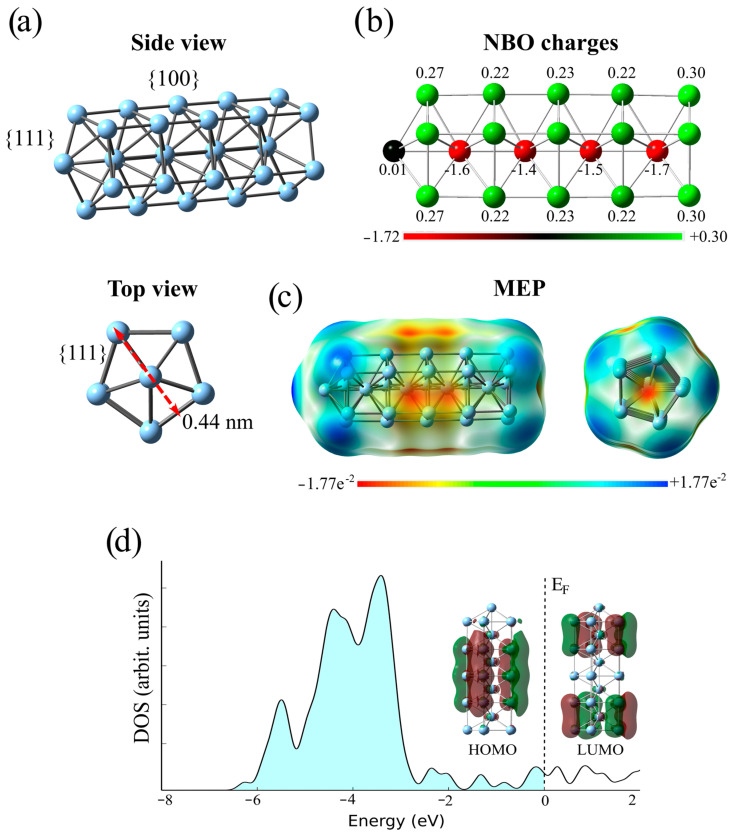
The optimized structure (**a**), natural bond orbital (NBO) charges (**b**), the molecular electrostatic potentials (MEP) (**c**), and the total density of states of the Ag30 cluster (**d**). Green and maroon denote positive and negative regions of the wavefunction.

First, the optimized structure of the Ag_30_ cluster is presented in Figure 5a (side and top views). The Ag_30_ cluster consists of five consecutive pentagonal rings with five silver atoms placed between these rings along the central C_5_-axis of symmetry. This implies that one silver atom is pressed into the cluster, and the other stays out (Figure 5). Interatomic distances between silver atoms vary from 2.78 to 3.00 Å depending on the position in the cluster. The distance between successive pentagons is about 3 Å, while their thickness is 4.4 Å (0.44 nm) as indicate with a red arrow (Figure 5c). It has been shown that {100} ends of silver nanowires are more reactive than their {111} facets [52].

According to the NBO charge analysis, the silver atoms in the cluster’s interior are negatively charged, whereas those on the cluster’s exterior are positively charged (Figure 5b). The exception is the silver atom that sticks out and is almost neutral. While the positive charges of silver atoms at the surface vary from +0.22 to +0.30 e^−^, those buried inside the cluster are much more negative, spanning from −1.40 to −1.72 e^−^. On the other hand, Figure 5c combines various colors to represent different MEP values. Red and blue represent the electron-rich (negative) and electron-deficient (positive) parts of the molecules, respectively, while green denotes areas with zero potential. The MEP shows that the electropositive regions are at the edges of the cluster (blue), while the negative parts are within the cluster (red), which is consistent with the NBO values. In addition, the MEP reveals electro-negative regions that surround the {100} surfaces.

The computed electronic structure of the Ag_30_ cluster illustrated through the density of state diagram indicates the metallic property of AgNW (Figure 5d). The electronic states near the Fermi level depicted via FMO’s wave-functions may suggest a smooth conductivity of AgNW.

To model an ideal graphene surface, the C_40_H_16_ cluster was constructed. To mimic the surface of graphene-oxide or reduced graphene-oxide, covered with epoxy and hydroxy groups, different molecular systems were employed, such as C_40_H_16_O_n_ and C_40_H_16_(OH)_n_ (n = 1–4), respectively. In addition, the C_40_H_16_O_2_(OH)_2_ cluster was utilized to describe the GO surface filled with both epoxy and hydroxyl groups.

**Figure 6:**
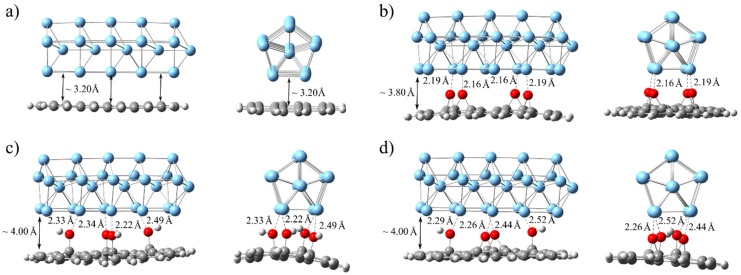
The optimized structures of Ag_30_@C_40_H_16_ (**a**), Ag_30_@C_40_H_16_O_4_ (**b**), Ag_30_@C_40_H_16_(OH)_4_ (**c**), and Ag_30_@C_40_H_16_O_2_(OH)_2_ (**d**) adducts as calculated at the B3LYP-D3/6-31G(d,p)/LANL2DZ level.

To estimate the binding strength between AgNW, modeled using the Ag_30_ cluster, and pristine graphene, as well as GO/rGO, we generated various Ag_30_@C_40_H_16_(O)_n_(OH)_n_ adducts and reoptimized them by preserving the Ag_30_ structure. The optimized structures of these adducts are presented in Figure 6. The short distances between oxygen atoms from epoxy and hydroxyl groups and Ag atoms (<2.5 Å) indicate strong interactions, according to Dannenberg et al. [53]. The interplane distance of 3.2 Å within the Ag_30_@C_40_H_16_ adduct is typical for stacking interactions, suggesting the dispersion nature of the interactions (Figure 6a). On the other side, geometrical parameters in Ag_30_@C_40_H_16_(O)_n_(OH)_n_ structures, with short O···Ag distances, point to the electrostatic and dispersion nature of bonding (Figure 6b–d).

The interaction energies, which represent interfacial bonding between the Ag_30_ cluster and various G- and rGO/GO-based systems, are calculated with the inclusion of Grimme’s dispersion correction (GD3). The counterpoise correction is used to remove the BSSE. All these energy values are listed in Table 1. The computed interaction value of −48.9 kcal/mol in the Ag_30_@C_40_H_16_ adduct suggests that there would be significant noncovalent binding between pristine graphene and AgNW. Since the C_40_H_16_ cluster has five condensed C_6_-rings along the C_2_-axis of symmetry, overlapping with four Ag_4_ fused rings of the Ag_30_ cluster, it could be roughly estimated that the interaction per C_6_-ring is −9.78 kcal/mol. For comparison, the energy of stacking interaction between two benzene rings is −2.78 kcal/mol [54]. On the other hand, including epoxy groups in graphene leads to a significant rise in interaction (binding) energy in Ag_30_@C_40_H_16_O_n_ adducts, reaching −92.9 kcal/mol for Ag_30_@C_40_H_16_O_4_. The dispersion energy loss caused by an interplane digression from 3.20 to 3.80 Å is compensated by strong (Ag···O) electrostatic interactions (Figure 6b). Considering the Ag_30_@C_40_H_16_(OH)_n_ adducts, it can be seen that the dangling OH groups interact with Ag atoms less strongly, which is reflected through longer Ag···O distances (Figure 6c). Including three OH groups into the graphene core overcomes the energy loss, impacted by larger interplane distance (~4.0 Å). The Ag_30_@C_40_H_16_(OH)_4_ adduct has the strongest binding energy between fragments, which is determined to be −75.1 kcal/mol (Table 1). The C_40_H_16_O_2_(OH)_2_ cluster is created to more closely resemble the GO surface coated with epoxy and hydroxyl groups. The interaction energy between Ag_30_ and C_40_H_16_O_2_(OH)_2_ fragments is calculated to be −81.9 kcal/mol, which is the value in between those calculated for the Ag_30_@C_40_H_16_O_4_ and Ag_30_@C_40_H_16_(OH)_4_ adducts. All these findings indicate that AgNW binds to the GO/rGO surface more firmly than it does to the ideal graphene surface.

The interaction energies were also calculated at the Hartree–Fock (HF) level, which does not account for the electron correlations. The contribution of the dispersion interaction based on the difference between the B3LYP-D3 and HF energies was estimated (Table 1). It can be observed that in the Ag_30_@C_40_H_16_ system, the dispersion interactions are the dominant binding forces. On the other hand, in both systems containing oxygen species, the electrostatic interactions prevail by increasing the number of epoxy/hydroxy groups. For the Ag_30_@C_40_H_16_O_2_(OH)_2_ complex, a slightly stronger interaction energy using the B3LYP-D3 method was obtained as compared to the pure HF which indicates a dominant electrostatic contribution to the overall binding.

According to zeta potential analysis, graphene oxide materials are negatively charged through a wide pH range [55]. The edge phenolic hydroxyl and carboxyl groups contribute more to the negative charge than the basal-plane hydroxyl and epoxy groups, according to FT-IR and UV-VIS spectroscopic investigations [55]. Thus, we introduced one or two negative charges into the graphene core via carboxyl and edge phenolic hydroxyl groups to estimate charge transfer behavior within Ag_30_/GO/rGO composites. Mulliken (Q_Mulliken_) and natural bond orbital (Q_NBO_) analyses are performed, and the results are listed in Table 2. The data indicate charge transfers from the graphene core to the Ag_30_ cluster considering mono- and di-anionic pristine graphene. According to the Q_NBO_ analysis on [Ag_30_@C_40_H_15_-COO]^−^ and [Ag_30_@C_40_H_15_-COO-O]^2−^ adducts, the Ag_30_ accepts electron densities of −0.59 and −0.67 e^−^, respectively. For rGO/GO filled with epoxy groups, the successive introduction of each epoxy group causes a reduction in electron transfer to the Ag_30_ cluster, making it even more positively charged in [Ag_30_@C_40_H_15_O_3_(O_4_)-COO]^−^ systems (Table 2). Such a trend is less pronounced in rGO/GO with hydroxyl groups. On the other hand, the Ag_30_ accepts electron densities from rGO/GO, ranging from −0.07 to −0.90 e^−^, in all cases involving double anionic species. For the graphene coated with both epoxy and hydroxyl groups, Ag_30_ remains positive in monoanionic adduct, but there is a tendency for electron transfer to the Ag cluster in double-anionic species (Table 2).

**Figure 7:**
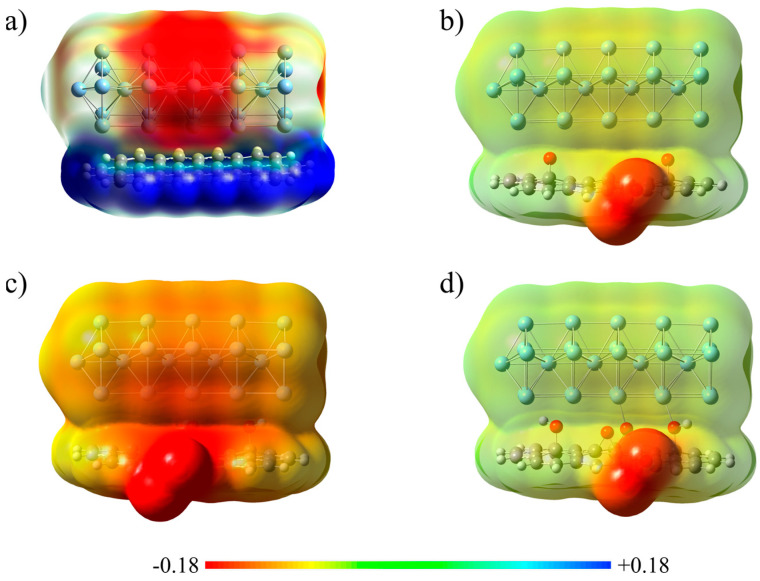
MEP plots of Ag_30_@C_40_H_16_ cluster (**a**), [Ag_30_@C_40_H_15_O_2_-COO]^−^ (**b**), [Ag_30_@C_40_H_14_(OH)_2_-COO-O]^2−^ (**c**), and [Ag_30_@C_40_H_15_O_2_(OH)_2_-COO]^−^ (**d**) adducts.

The electron-accepting behavior of Ag_30_ within Ag_30_@rGO/GO composites is also proved by analyzing molecular electrostatic potentials (MEPs) of relevant species. As already depicted in Figure 5c, the different colors signify different MEP values: red and blue show negative and positive parts of the nanowire, while the green color represents zero potential regions. While the MEP of Ag_30_@C_40_H_16_ shows that the electropositive is the graphene core and negative is the region that surrounds the Ag_30_ (Figure 7a), inside the [Ag_30_@C_40_H_15_O_2_-COO]^−^ system, the Ag_30_ fragment is slightly negatively charged (yellowish; Figure 7b). At variance, Ag_30_ exhibits more negatively charged surroundings (reddish) in the [Ag_30_@C_40_H_14_(OH)_2_-COO-O]^2−^ adduct, indicating higher transmission of electrons from the rGO to the Ag_30_ (Figure 7c). Like the previous case, in the [Ag_30_@C_40_H_15_O_2_(OH)_2_-COO]^−^ system, the Ag_30_ is moderately negatively charged (Figure 7d).

All these results indicate that AgNWs would be stabilized via passivation with rGO/GO, owing to the charge transfer from the graphene core to the AgNWs.

While UV-Vis and FTIR indicated establishing interaction between GO and AgNWs, Raman spectra showed “healing” of GO sheets and suggested improvement to the structural order of sp^2^ structure in GO-AgNW composites. Theoretical analysis of GO:AgNWs composites consider GO with different levels of oxidation and study its interaction with AgNWs. These results suggest charge transfers from the graphene core to the Ag_30_ cluster. The primary source of interfacial polarization is the electrical conductivity differential between the two materials at the interface, which allows charge redistribution across the contact surface.

All these results suggest that GO-AgNWs composites should possess higher electron density at the interface compared to GO or rGO. Thus, in the further part of the manuscript, we analyzed the morphology of these composites in the form of free-standing films and explored their ability to block the propagation of EMWs in the frequency region of 150 KHz–18 GHz.

### 2.3. Analysis of Free-Standing Composites

**Figure 8:**
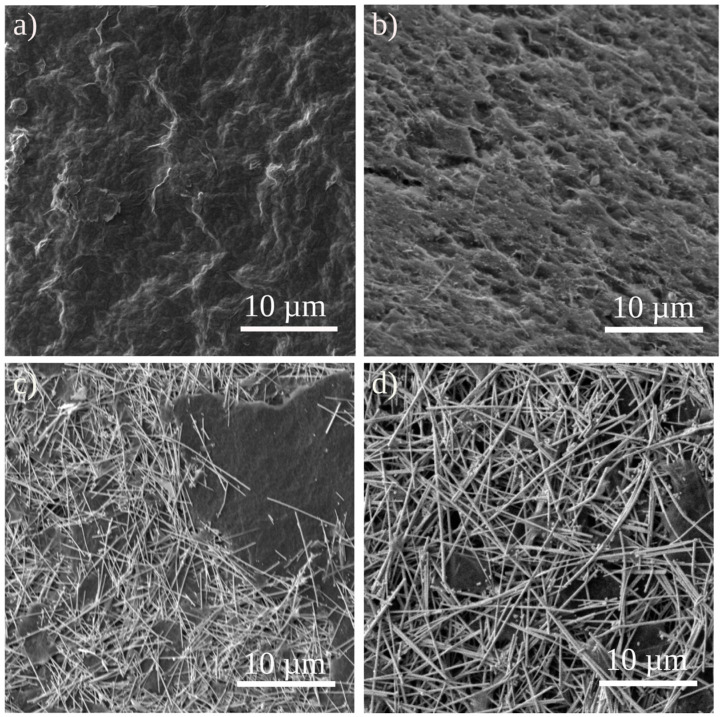
Top and side views of free-standing films recorded using SEM of GO (**a**), GO-AgNWs 5:5 (**b**), GO-AgNWs 3:7 (**c**), and GO-AgNWs 1:9 (**d**).

Figure 8 shows top-view SEM images of free-standing GO and GO-AgNWs 5:5, 3:7, and 1:9 composites. Sheet-like morphology is displayed in Figure 8a, where GO sheets create a wavy surface. In the cross-section SEM images, the hollow interface between the layers is detected (Figure S6a, Supporting Information). In the case of GO-AgNWs 5:5 composite (Figure 8b), the surface of the film is wavier and rough, with holes and sporadically distributed rods (AgNWs) imbedded in sheets (Figure S6b, Supporting Information). With increased content of AgNWs in composites (Figure 8c,d), rods are more densely distributed over the surface of the composite, in the top-view SEM images. Cross-section SEM images showed that AgNWs are equally distributed among GO layers (Figure S6c,d, Supporting Information). The average thickness of GO was 13.34 μm ± 1.07 μm, 9.94 μm ± 0.93 μm for GO-AgNWs 5:5, 8.94 μm ± 1.05 μm for GO-AgNWs 3:7, and 12.39 μm ± 1.19 μm for GO-AgNWs 1:9 (Figure S6, Supporting Information).

SEM images of GO and GO-AgNW composites showed that sheets- and rod-like objects are equally distributed on the surface and inside of free-standing composites. Although the same amount of nanomaterials was used to obtain free-standing films, the average thickness varied from 13.34 μm for GO to 9.94 μm for GO-AgNWs 5:5. The same mass of nanomaterials was used for each free-standing sample, 15 mg in total. Differences in the thicknesses result from the difference in the nanomaterials assembly due to their different geometry, flexibility, and shape.

**Figure 9:**
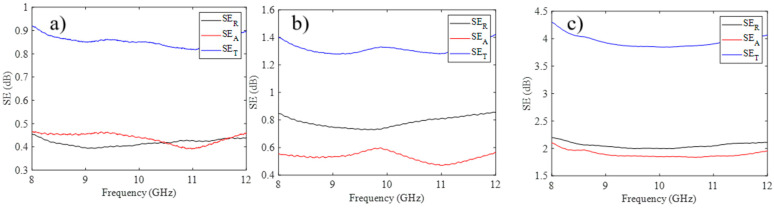
SE_T_, SE_A_, and SE_R_ values for GO-AgNWs 5:5 (**a**), GO-AgNWs 3:7 (**b**), and GO-AgNWs 1:9 (**c**), measured in the frequency range of 8–12 GHz.

The EMI shielding effectiveness of all composites is also analyzed. The shielding effectiveness of the GO sample was reported earlier [56]. It was shown that GO provides negligible shielding effectiveness against electromagnetic waves with frequencies of 8–12 GHz. The total shielding effectiveness, reflective shielding effectiveness, and absorptive shielding effectiveness of the GO-AgNWs composites with different GO to AgNWs mass ratios are shown in Figure 9. Compared to GO, all the composites that contained AgNWs showed enhanced SE (Figure 9), from 0.9 dB to 4.5 dB as compared to other studies, where GO/AgNWs composites showed shielding efficacy between 35.5 and 55.16 dB. This difference in the shielding effectiveness values could be due to the thickness of the composites which is approximately 30 μm or higher [36,37,57]. Moreover, differences in GO defects and the morphological properties of AgNWs such as the length and width can also contribute to the difference in the shielding values. AgNWs possess higher electrical conductivity compared to GO. The composites with higher concentrations of AgNWs provide higher values of total shielding effectiveness and reflective shielding effectiveness (SE_T_ from 0.9, 1.4, to 4.0 and SE_R_ 0.4, 0.8 and 2 dB, for GO-AgNWs 5:5, GO-AgNWs 3:7, and GO-AgNWs 1:9, respectively). With higher amounts of AgNWs in composites, slightly higher values of SE_A_ are measured, increasing from 0.4 dB, 0.5 dB, to 1.9 dB (Figure 9) along with AgNWs content. In previous studies, EMW shielding was studied for AgNWs [58], GO-AgNWs [57], and GO-AgNWs-GO composites. These studies indicated that AgNWs improve the shielding effectiveness of GO and was explained by the increase in electrical conductivity [59]. The calculated total density of states (TDOSs) of C_40_H_16_O_2_(OH)_2_ and Ag_30_@C_40_H_16_O_2_(OH)_2_ molecular structures, representing GO and GO/AgNWs, indicated that the bandgap is much reduced in the GO/AgNW adduct (Figure S5). The sheet resistance of samples was measured using the 4-point probe method, and the following values were obtained: for GO-AgNWs 5:5 was 5.2 ± 0.2 Ω/□, for GO-AgNWs 3:7 was 5.0 ± 0.6 Ω/□, and for GO-AgNWs 1:9 was 17.7 ± 0.6 Ω/□. In the case of GO, sheet resistance could not be measured. Thus, GO-AgNWs 5:5, GO-AgNWs 3:7, and GO-AgNWs 1:9 samples are electrically conductive, while GO was non-conductive. The structural and theoretical analyses showed that AgNWs accept electron density transferred from the graphene sheets. The charge accumulation at the interface validates the interfacial polarization due to the difference in electrical conductivity between AgNW and GO, resulting in a conductive network and improving the EMI shielding behavior of these composite materials [60]. All composites showed the ability to reflect and absorb EMWs, but the main shielding mechanism is due to reflection, which agrees with electrical conductivity measurements. Composites with higher concentrations of AgNWs provide higher values of the total and reflective shielding effectiveness.

Although this and earlier studies indicated that GO-AgNWs-based composites possess the ability to block the propagation of EMW in various frequency ranges, future application of these materials will be limited by their sensitivity to the following properties:

- Thermal stability below 150 °C, considering that TGA showed the structure of GO, and GO-AgNWs are changing the compositions above (Figure 2), while AgNWs are melting at this temperature [61];
- UV light exposure [62];
- Exposure to atmospheric conditions, such as oxygen, humid, and sulfurous oxides, could all lead to chemical changes in AgNWs [63].

Apart from ideal models of graphene and graphene oxide, in the real samples, various defects and impurities such as residual reagents could be present which can affect the shielding efficiency of the materials. While oxygen in GO sheets improves the EMI SE, other defects such as holes create ruptures in the π cloud [64]. These cracks are regions where EMWs could be transmitted, reducing EMI SE. In the process of AgNWs synthesis, the polymer is used to stabilize and direct the wire production [65], which remains after cleaning of the AgNWs surface and affects contact between AgNWs [66]. This impurity is another factor that could lower EMI SE, along with Ag_2_O or Ag_2_S that could be formed easily upon Ag reaction with atmospheric O_2_ and H_2_S [63].

## 3. Materials and Methods

### 3.1. Materials

GO is produced using a chemical reaction called “modified Hummer’s method” [67]. Herein, graphite powder (1 g, type Z-346 KS6, TIMREX^®^, Bodio, Switzerland) is mixed with concentrated H_2_S0_4_ (23.3 mL, Carl Roth, Karlsruhe, Germany) at 4 °C. During mixing, KMnO_4_ (3 g, Merck, Darmstadt, Germany) is added carefully in small portions to the reaction mixture. The mixture is stirred for 30 min to stabilize and homogenize. Then, the reaction mixture is gradually heated to 40 °C. In the next step, demineralized water is added (50 mL), and the temperature is maintained for 30 min. Afterwards, the temperature is increased to 90 °C and kept for 15 min. The reaction was stopped by removing the mixture from the hot plate and cooled to room temperature. In the following steps, GO is cleaned from reagents according to the procedure described in a previously published paper [67]. The resulting GO dispersion is used to prepare composites with AgNWs and free-standing films.

AgNWs are synthesized in a “polyol” procedure [52]. First, 3.7029 g of polyvinylpyrrolidone 40 (PVP40, average mol wt 40,000, Sigma Aldrich, St. Louis, MO, USA) is dissolved in ethylene glycol (EG, purity (GC) ≥ 99.5%, Sigma Aldrich, St. Louis, MO, USA) by heating at 160 °C for 30 min. Then, 150 µL of NaCl (ACS reagent, ≥99.0%, Sigma Aldrich, St. Louis, MO, USA) solution in EG (0.15 mM) is added. The same volume of FeCl_3_ (anhydrous, VWR International GmbH, Darmstadt, Germany, in EG, 0.15 mM) is added. The solution of AgNO_3_ (ACS reagent, ≥99.0%, Sigma Aldrich, St. Louis, MO, USA) in EG is prepped at 0.15 M concentration. This solution is added to the reaction very slowly in a portion of 10 μL until the total volume reached 40 mL. The mixture is stirred for 8 h at 160 °C. The resulting product is washed with ethanol (96 vol.%, Betahem, Belgrade, RS, Serbia) and stored in this solvent.

Composites based on GO and AgNWs are obtained using the previously described procedure [27,68]. Dispersions of GO (1 mg mL^−1^) in water and AgNWs (1 mg mL^−1^) in 96 vol% ethanol are mixed in different volume ratios. The volume and mass ratios in % of GO to AgNWs are 5:5, 4:6, 3:7, 2:8, and 1:9, respectively. In the following part of the manuscript, samples are referred to as GO-AgNWs 5:5, GO-AgNWs 4:6, GO-AgNWs 3:7, GO-AgNWs 2:8, and GO-AgNWs 1:9, depending on their content of each nanomaterial. After each mixture is stirred for 30 min using a mechanical stirrer, at a speed of 50 rpm, homogenous dispersions are obtained. These dispersions are transformed into free-standing films by pouring 15 mL of each dispersion into a vacuum filtration system equipped with a hydrophilic membrane filter (IsoporeTM membrane filter, pore size 0.22 μm, GTTP02500, polycarbonate, Merk, Darmstadt, Germany). By applying vacuum, equal pressure is distributed over the whole membrane surface allowing for the uniform spreading of GO-AgNWs composites. After the liquid has passed the membrane, composites are left on the membrane surface and peeled off after drying.

One set of samples is chemically reduced using L-ascorbic acid (L-AA). Free-standing films are immersed in an L-AA water solution (15.14 mM). To reach acidic pH, 100 μL of 5 M HCl is added. Free-standing films are placed in a solution of L-AA and HCl and heated at 85 °C for 8 h. Afterwards, samples are gently placed in demineralized water and dried. These samples are named rGO-AgNWs 5:5, rGO-AgNWs 4:6, rGO-AgNWs 3:7, rG-AgNWs 2:8, and rGO-AgNWs 1:9.

### 3.2. Methods

UV-Vis, Raman, and infrared spectroscopies are used to investigate GO, AgNWs, and GO-AgNWs structures, as well as interactions between nanomaterial surfaces. To investigate optical properties and establish interaction between the 2 nanomaterials, GO and AgNWs, UV-Vis spectra are recorded using an LLG-uniSPEC 2 spectrophotometer (Lab Logistic group, Mekenheim, Germany). GO, AgNWs, and composites dispersions are diluted to a 0.03125 mg mL^−1^ concentration. UV-Vis spectra are recorded in the range of 190–800 nm, with a resolution of 2 nm. The Nicolet iS20 spectrometer (Thermo Scientific, Waltham, MA, USA) is utilized for FTIR analysis. The sample is applied directly onto the diamond ATR crystal, with spectra recorded at a resolution of 4 cm^−1^ and a scan rate of 16 scans/spectrum. A DXR Raman microscope (Thermo Fisher Scientific, Waltham, MA, USA) is used to collect Raman spectra of GO composites. For excitation, a laser with a 532 nm wavelength is used. For each sample, 3 different spots were selected, and spectra were recorded and used to calculate intensity ratios between D and G bands. The laser power is 2 mW, and the acquisition time is set to 10 × 10 s.

To investigate the efficacy of chemical reduction and the thermal stability of GO-AgNW composites, a Mettler Toledo TGA/DSC 1 instrument (Mettler Toledo, Columbus, OH, USA) is used. Approximately 3 mg of each sample is used for TGA analysis. The thermal behavior is investigated from 40 °C to 750 °C under an N_2_ flow at a rate of 20 mL min^−1^. The heating rate is set to 5 K min^−1^. For each GO-AgNW composite, the measurements are repeated two times.

The Gaussian 09 software package is used for all the calculations [69]. The structural parameters, electronic properties, and interaction energies of the AgNW/G/rGO composites are investigated using density functional theory (DFT) modeling. The Ag_30_ cluster is constructed using a silver nanowire structure with pentagonal cross-sections with ends terminated by {111} facets and side surfaces representing {100} facets [52]. To model pristine graphene (G), we employed the C_40_H_16_ cluster, while for the graphene-oxide/reduced graphene-oxide (GO/rGO), various clusters are used, such as C_40_H_16_O_n_, C_40_H_16_(OH)_n_ (n = 1–4), and C_40_H_16_O_2_(OH)_2_. The ground-state geometries of different Ag_30_@C_40_H_16_O_n_(OH)_n_ (n = 0–4) adducts are optimized using the B3LYP-D3 function [70,71,72], in combination with 6–31G(d,p) basis set for light atoms [73], and LANL2DZ basis set with pseudo potentials for Ag atoms [74]. The silver atoms of the Ag_30_ cluster are frozen during the optimization process to retain the pentagonal nanowire crystal structure, whereas all the atoms of G and rGO clusters are allowed to relax. The non-bonding interactions in the AgNW/G and AgNW/rGO/GO systems are presented as the interaction energies (E_int_), and counterpoise corrections are used to eliminate the basis set superposition error (BSSE) [75]. The following equations are used in this calculation process:

ΔE_int_ = E_Ag30_ + E_rGO(G)_ − E_composite_ (1)

ΔE_int_^CP^ = ΔE_int_ − E_BSSE_ (2)

The charge distributions on these composites are depicted through Mulliken charges and natural bond orbitals (NBO) analysis, as well as molecular electrostatic potentials (MEPs). All these calculations are performed in the gas phase.

A scanning electron microscope (SEM) is used to investigate the morphology of free-standing GO, rGO, and GO-AgNWs composites, as well as GO and AgNWs. A high-resolution scanning electron/focused ion beam (dual-beam) microscope Tescan^®^ LYRA 3 FEG/XMH(Tescan group a.s. Brno, Czech Republic) SEM was used. Samples are fixed on the surface of the support using double-sided carbon tape. A secondary electron detector is used to capture the images. The acceleration voltage was 10 kV, and measurements are conducted in a high-vacuum. The cross-section of composites was analyzed using the software ImageJ 1.x to estimate the sample thickness. Thickness was measured at 5 positions, and an average value was calculated. To estimate the GO% of O, SEM-EDS (INCAx-act LN2-free analytical silicon drift detector of characteristic X-rays with PentaFET^®^ Precision a (Oxford Instruments, Oxfordshire, UK) with a TESCAN Mira3 XMU, SE detector) was used and obtained mas% of C was 54.3, O was 43.6 mas%, and S was 1.1 mas% which is residual from synthesis (Figure S7, Supporting Information). Average AgNWs were calculated using SEM images and the ImageJ software. The average diameter was 73 ± 12 nm, and the average length was 4.03 μm ± 0.62 μm, and the average aspect ratio was 1:55.

To investigate shielding effectiveness, GO-AgNW composites are prepared as free-standing films. Microwave experimental setup is used to measure the shielding properties of low-thickness, two-dimensional material [76]. For GO, rGO, GO-AgNWs 5:5, GO-AgNWs 4:6, GO-AgNWs 3:7, GO-AgNWs 2:8, and GO-AgNWs 1:9, as well as reduced form of composites, the amplitude of the transmission coefficient (S_21_) is measured. A vector network analyzer (VNA, compact Streamline 5008A, Keysight Technologies^®^, Santa Rosa, CA, USA) is used to measure the S-parameters. The frequency range is set from 150 kHz to 18 GHz considering 801 frequency points. Coaxial RF cables are used to connect the VNA to the test cell. The material under test (MUT), is sandwiched between the two coaxial apertures. Consequently, the transmitted microwave signal between the two coaxial apertures is affected by the electromagnetic properties of the MUT. In addition, microwave reflections occur at the MUT interface. Measurement configurations include thru (empty structure), reference (aluminum), and GO-based samples. Samples are inserted between two cellulose sheets with 90 µm thickness. A conventional short-open-load-thru (SOLT) coaxial calibration is applied at the outputs of the coaxial cables using an Anritsu^®^ TOSLKFOA-43.5 reference K-coaxial calibration kit (Anritsu, Kanagawa Prefecture, Japan). An amplitude normalization to the thru connection (direct connection of the coaxial apertures) is considered to remove residual systematic errors.

To estimate the shielding effectiveness of GO and GO-AgNWs composites, Equations (3)–(5) were used [77]:

SE_T_ = −S_21_ dB (3)

SE_R_ = −10log(1 − |S_11_|^2^) (4)

SE_A_ = −10log(|S_21_|^2^/(1 − |S_11_|^2^)) (5)

where SE_T_ is the shielding effectiveness due to transmission; SE_A_ is the result of EMW dissipation; SE_R_ is due to the wave reflection (SE_R_); S_11_ is the reflection coefficient, and S_21_ is the transmission coefficient. A vector network analyzer was used to measure S_11_ and S_21_ coefficients.

Electrical resistivity was measured using a 4-point probe Jandel RM3000+ (Leighton Buzzard, UK) test unit. The distance between probes is 1 mm. The samples of GO-AgNWs composites were analyzed at 3 different locations, and the average values were calculated.

## 4. Conclusions

Composites based on GO and AgNWs are produced in different mass ratios of each component. The interaction between the two different nanomaterials is studied using experimental (Raman, UV-Vis, FTIR, and TGA) and theoretical approaches (DFT). By analyzing the Mulliken and NBO atomic charges, as well as MEPs, it is revealed that charge transfer occurs from GO to AgNWs, resulting in the redistribution of charges across the interface. As a result, a conductive network is created, improving the EMI shielding properties. Our results confirm the establishment of electron transfer between GO and AgNWs, leading to an improvement of the shielding effectiveness of the composites.

## Figures and Tables

**Table 1:** The interaction energies (kcal/mol) in various AgNW/G/GO/rGO adducts, all calculated at the B3LYP-D3//HF/6-31G(d,p)/LANL2DZ level. Energy values are corrected for BSSE.

Species	Interaction Energy (kcal/mol)	
	B3LYP-D3	HF
Ideal graphene		
Ag_30_@C_40_H_16_	−48.9	31.8
GO with epoxy groups		
Ag_30_@C_40_H_16_O	−49.4	−6.6
Ag_30_@C_40_H_16_O_2_	−60.6	−35.9
Ag_30_@C_40_H_16_O_3_	−77.7	−71.3
Ag_30_@C_40_H_16_O_4_	−92.9	−101.9
GO with hydroxy groups		
Ag_30_@C_40_H_16_(OH)	−42.5	-
Ag_30_@C_40_H_16_(OH)_2_	−46.6	−14.3
Ag_30_@C_40_H_16_(OH)_3_	−58.1	−39.4
Ag_30_@C_40_H_16_(OH)_4_	−75.1	−68.6
GO with epoxy and hydroxyl groups		
Ag_30_@C_40_H_16_O_2_(OH)_2_	−81.9	−80.0

**Table 2:** Mulliken and NBO charges analysis (electron units) and dipole moments (Debye) for various Ag_30_@rGO/GO anionic species.

Species	Q_Mulliken_	Q_NBO_	Dipole Moment
Ideal graphene			
[Ag_30_@C_40_H_15_-COO]^−^	−0.64	−0.59	13.28
[Ag_30_@C_40_H_15_-COO-O]^2−^	−0.86	−0.67	8.31
rGO with epoxy groups			
[Ag_30_@C_40_H_15_O-COO]^−^	−0.19	−0.28	15.38
[Ag_30_@C_40_H_15_O_2_-COO]^−^	−0.30	−0.42	15.57
[Ag_30_@C_40_H_15_O_3_-COO]^−^	+0.17	+0.16	14.55
[Ag_30_@C_40_H_15_O_4_-COO]^−^	+0.47	+0.54	17.93
[Ag_30_@C_40_H_14_O-COO-O]^2−^	−0.75	−0.83	18.62
[Ag_30_@C_40_H_14_O_2_-COO-O]^2−^	−0.65	−0.73	20.71
[Ag_30_@C_40_H_14_O_3_-COO-O]^2−^	−0.46	−0.46	15.36
[Ag_30_@C_40_H_14_O_4_-COO-O]^2−^	−0.14	−0.07	19.16
rGO with hidroxy groups			
[Ag_30_@C_40_H_15_(OH)-COO]^−^	−0.27	−0.26	16.15
[Ag_30_@C_40_H_15_(OH)_2_-COO]^−^	−0.29	−0.21	16.29
[Ag_30_@C_40_H_15_(OH)_3_-COO]^−^	−0.23	−0.18	15.43
[Ag_30_@C_40_H_15_(OH)_4_-COO]^−^	−0.05	+0.19	20.83
[Ag_30_@C_40_H_14_(OH)-COO-O]^2−^	−0.78	−0.71	19.89
[Ag_30_@C_40_H_14_(OH)_2_-COO-O]^2−^	−0.80	−0.70	21.05
[Ag_30_@C_40_H_16_(OH)_3_-COO-O]^2−^	−0.90	−0.68	17.75
[Ag_30_@C_40_H_14_(OH)_4_-COO-O]^2−^	−0.85	−0.55	19.42
rGO with epoxy and hydroxyl groups			
[Ag_30_@C_40_H_15_O_2_(OH)_2_-COO]^−^	+0.16	+0.42	19.82
[Ag_30_@C_40_H_14_O_2_(OH)_2_-COO-O]^2−^	−0.44	−0.17	21.54

## Data Availability

Datasets analyzed in the current study are available in the Zenodo repository (https://doi.org/10.5281/zenodo.14173026).

## Supplementary Materials

The supporting information can be downloaded at: https://www.mdpi.com/article/10.3390/ijms252413401/s1.
