# Supplementary material for: Study of Graphene Oxide and Silver Nanowires Interactions and Its Association with Electromagnetic Shielding Effectiveness
Source: Int J Mol Sci. 2024 Dec 13;25(24):13401. doi: 10.3390/ijms252413401 (PMC11677061; doi:10.3390/ijms252413401)
Supplement: Supplementary file 1 [file ijms-25-13401-s001.zip › ijms-3347095-supplementary.pdf]

Supporting Information

Article

Study of graphene oxide and silver nanowires interactions and its association with electromagnetic shielding effectiveness

Mila Milenkovic[1], Warda Saeed[2], Muhammad Yasir[2]*, Dusan Sredojevic[1], Milica Budimir[1], Andjela Stefanovic[1], Danica Bajuk–Bogdanovic[3], Svetlana Jovanović[1]*

[1] Vinča Institute of Nuclear Sciences-National Institute of the Republic of Serbia, University of Belgrade, P.O. Box 522, 11000 Belgrade, Serbia; mila.milenkovic@vin.bg.ac.rs, dusredo@vin.bg.ac.rs, budimir@vin.bg.ac.rs, svetlanajovanovic@vin.bg.ac.rs

[2] Carl von Ossietzky Universität Oldenburg, 26129 Oldenburg, Germany; muhammad.yasir@uni-oldenburg.de, warda.saeed@uni-oldenburg.de

[3] University of Belgrade, Faculty of Physical Chemistry, Studentski trg 12-16, Belgrade 11158, Serbia; danabb@ffh.bg.ac.rs

* Correspondence: S.J. svetlanajovanovic@vin.bg.ac.rs; Vinča Institute of Nuclear Sciences-National Institute of the Republic of Serbia, University of Belgrade, P.O. Box 522, 11000 Belgrade, Serbia, M.Y. muhammad.yasir@uni-oldenburg; Carl von Ossietzky Universität Oldenburg, 26129 Oldenburg, Ger-many;

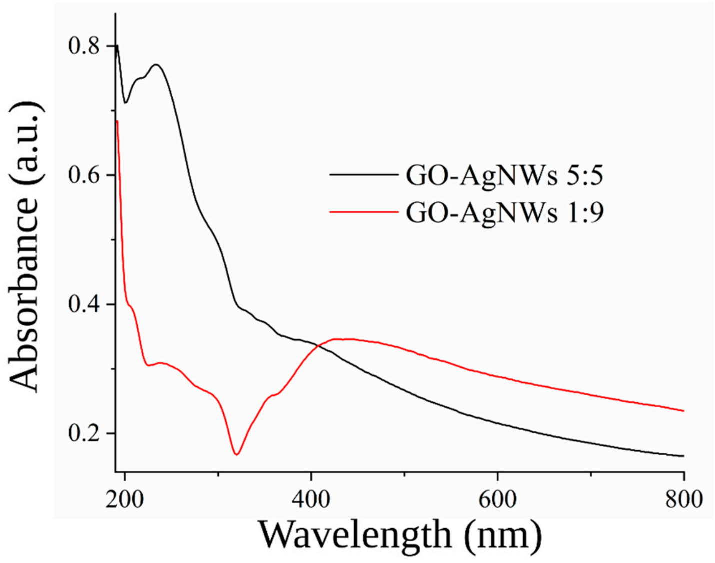

Figure S1. UV-Vis spectra of GO-AgNWs 5:5 (black) and GO-AgNWs 1:9 (red curve).

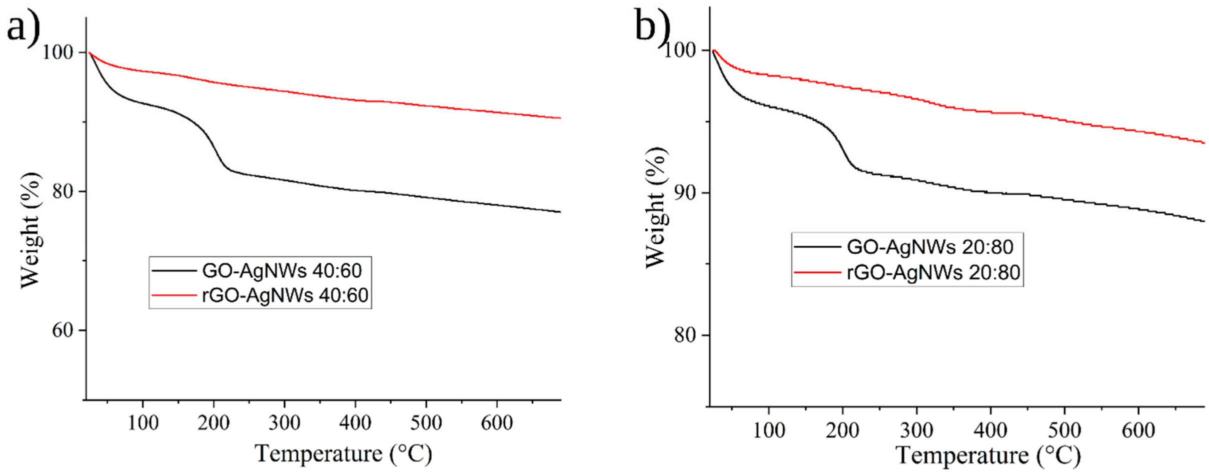

Figure S2. Thermograms GO-AgNWs 4:6 and rGO-AgNWs 4:6 (a), GO-AgNWs 2:8 and rGO-AgNWs 2:8 (b).

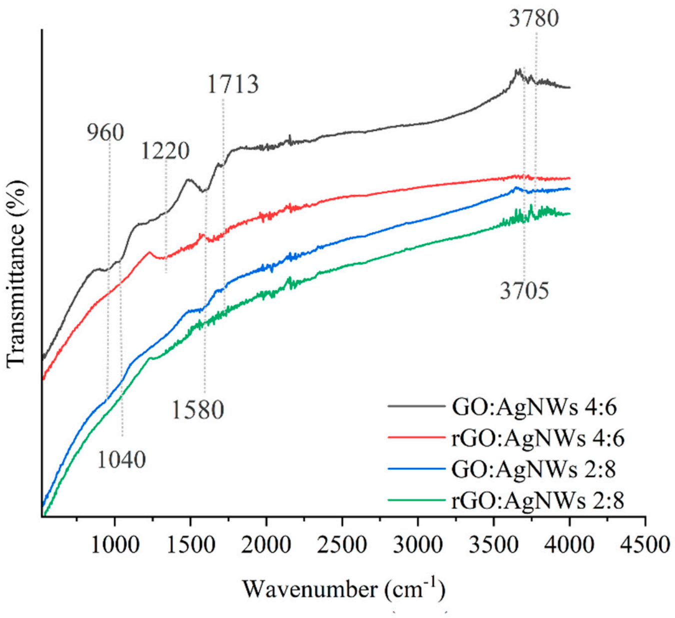

Figure S3. FTIR spectra of GO-AgNWs 4:6, rGO-AgNWs 4:6, GO-AgNWs 2:8, and rGO-AgNWs 2:8.

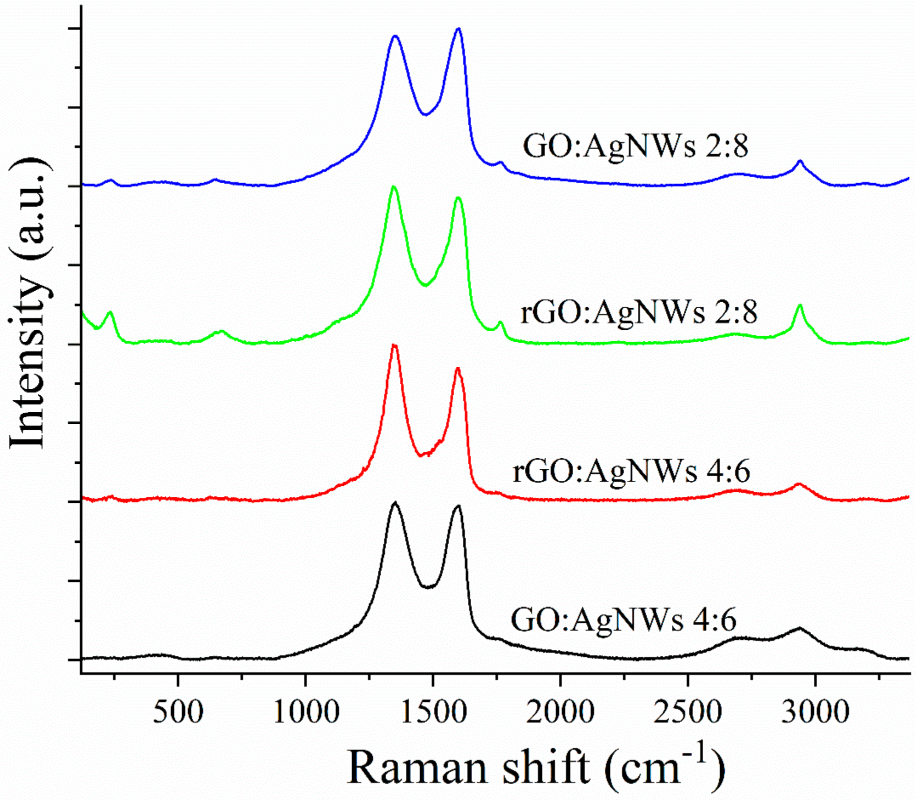

Figure S4. Raman spectra of GO-AgNWs 4:6, rGO-AgNWs 4:6, GO-AgNWs 2:8, and rGO-AgNWs 2:8.

Table S1. Analysis of Raman spectra: positions of D, G bands and calculated values for I_D/I_G ratios for GO, rGO, and GO-AgNWs composites.

Sample	D (cm^{-1})	G (cm^{-1})	I_D/I_G
GO	1346	1594	1.00±0.11
GO-AgNWs 5:5	1354	1599	1.02±0.08
GO-AgNWs 4:6	1352	1596	1.03±0.05
GO-AgNWs 3:7	1349	1595	1.00±0.07
GO-AgNWs 2:8	1352	1600	0.95±0.06
GO-AgNWs 1:9	1348	1595	0.92±0.08
rGO	1347	1597	1.21±0.13

rGO-AgNWs 5:5	1350	1602	1.22±0.08
rGO-AgNWs 4:6	1350	1601	1.16±0.03
rGO-AgNWs 3:7	1351	1602	1.19±0.13
rGO-AgNWs 2:8	1348	1602	1.06±0.09
rGO-AgNWs 1:9	1349	1595	0.89±0.10

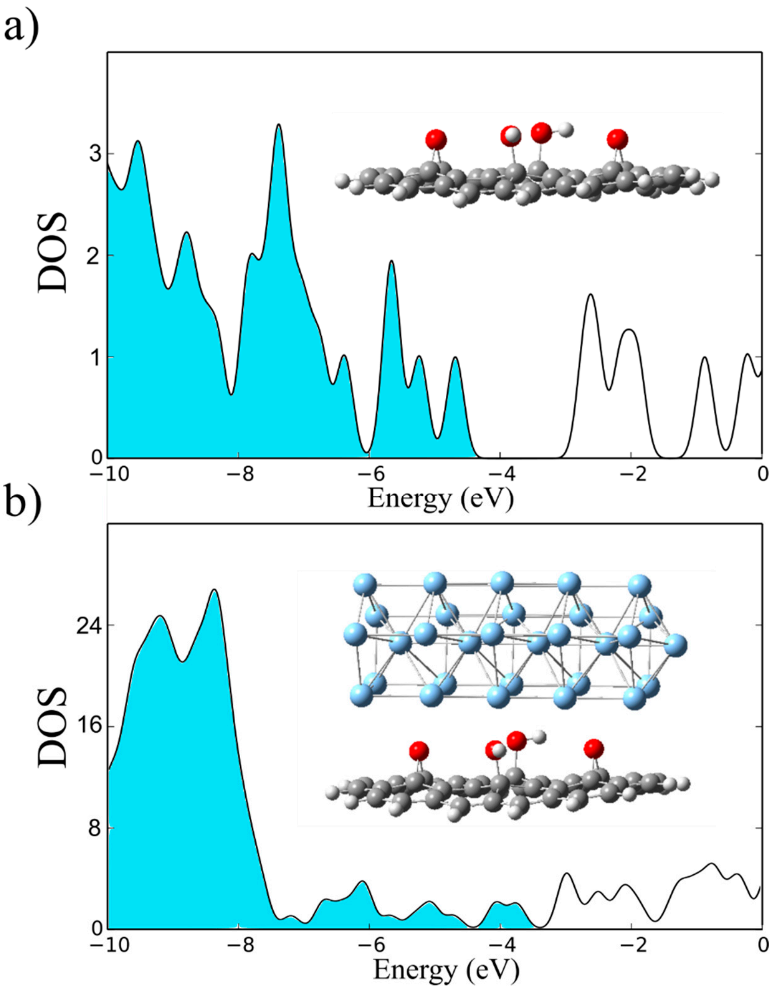

Figure S5. Total density of states (TDOS) diagrams of a) $C_{40}H_{16}O_2(OH)_2$ and b) $Ag_{30}@C_{40}H_{16}O_2(OH)_2$.

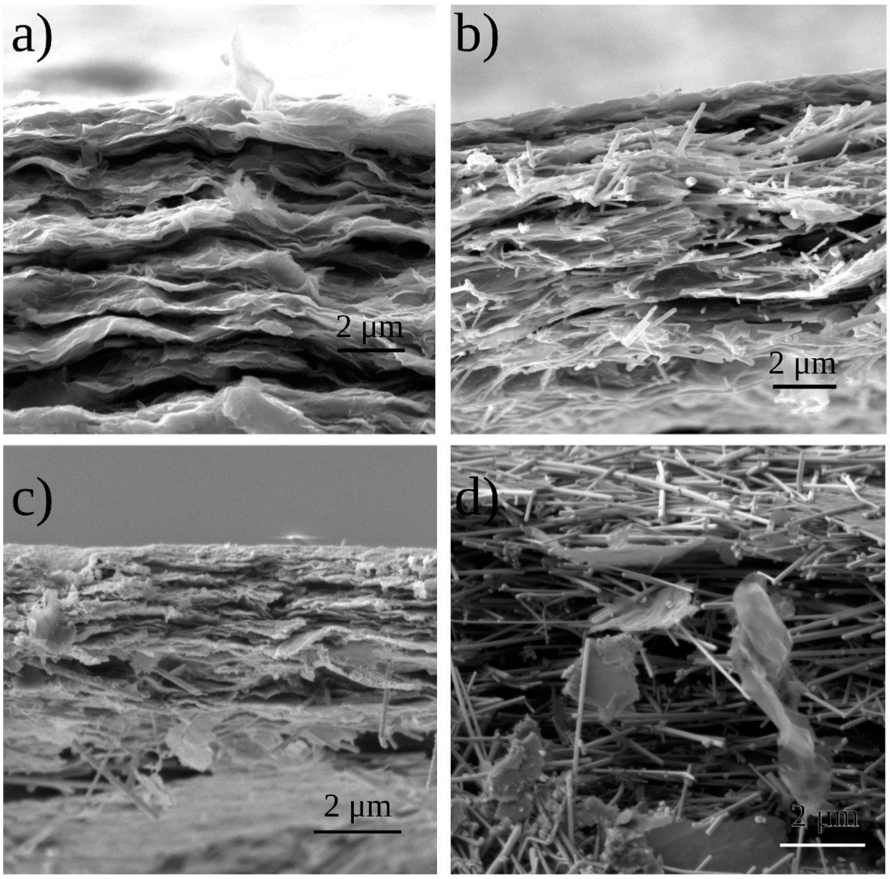

Figure S6. Cross-section SEM images of GO (a), GO-AgNWs 5:5 (b), GO-AgNWs 3:7 (c), and GO-AgNWs 1:9 samples (d).

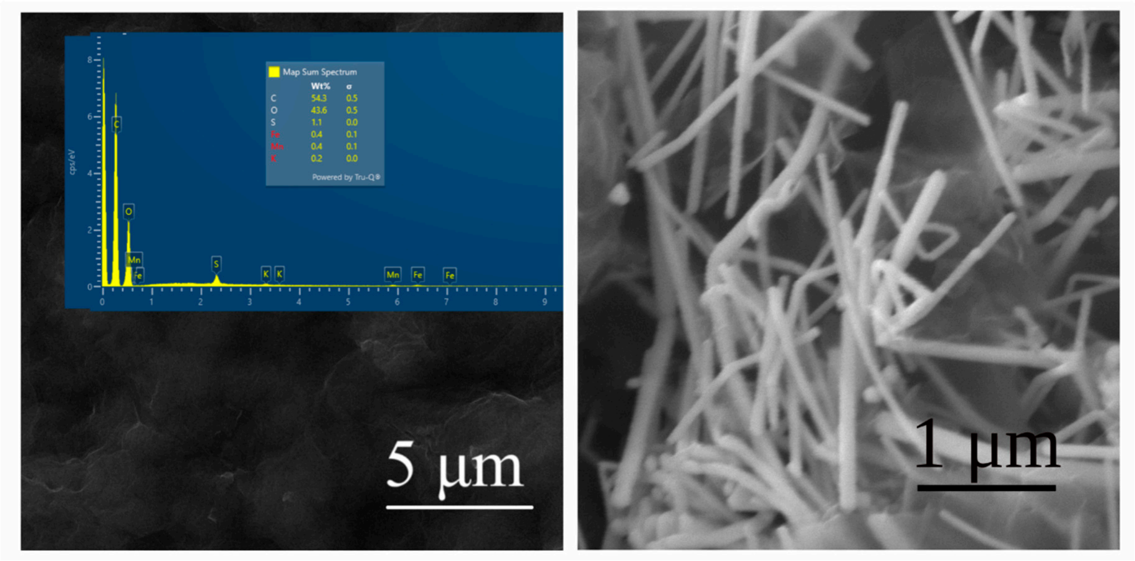

(a) (b)

Figure S7. SEM images of GO with EDS spectrum (a), and AgNWs (b).
